# The Action of JAK/STAT3 and BMP/HJV/SMAD Signaling Pathways on Hepcidin Suppression by Tucum-do-Cerrado in a Normal and Iron-Enriched Diets

**DOI:** 10.3390/nu12051515

**Published:** 2020-05-22

**Authors:** Sandra Fernandes Arruda, Larissa Valadares Ramos, Júlia Lima de Alencar Barbosa, Natália Aboudib Campos Hankins, Pedro Augusto Matos Rodrigues, Marcela de Sá Barreto da Cunha

**Affiliations:** 1 Postgraduate Program in Human Nutrition, Faculty of Health Sciences, Campus Universitário Darcy Ribeiro, Universidade de Brasília, Brasília 70910-900, Brazil; lari_vr16@hotmail.com (L.V.R.); nataliaaboudib@gmail.com (N.A.C.H.); desa.marcela@gmail.com (M.d.S.B.d.C.); 2 Department of Nutrition, Faculty of Health Sciences, Campus Universitário Darcy Ribeiro, Universidade de Brasília, Brasília 70910-900, Brazil; julia_alencar95@hotmail.com; 3 Institute of Physics, Campus Universitário Darcy Ribeiro, Universidade de Brasília, Brasília 70910-900, Brazil; pedro@unb.br

**Keywords:** tucum-do-cerrado, hepcidin, pSMAD1/5/8, pSTAT3, iron

## Abstract

The Brazilian savanna fruit, tucum-do-cerrado (*Bactris setosa* Mart.) reduces hepatic hepcidin levels. Therefore, we investigated the effect of tucum-do-cerrado on the TfR/HFE and/or BMP/HJV/SMAD and JAK/STAT pathways, in normal and excess iron conditions. Rats were treated with: control diet (CT); control diet +15% tucum-do-cerrado (Tuc); iron-enriched diet (+Fe); or iron-enriched diet +15% tucum-do-cerrado (Tuc+Fe). Tucum-do-cerrado (Tuc) decreased hepatic Hamp and *Hjv* mRNA levels but did not alter *Bmp6*, *Smad7*, *Tfr1*, and *Hfe* mRNA levels; pSMAD1/5/8 and pSTAT3 protein levels; labile iron pool (LIP); and inflammatory biomarkers, compared to the CT group. The iron-enriched diet increased *Hamp* mRNA levels, as well as pSMAD1/5/8 and pSTAT3 protein levels, while no difference was observed in *Hjv*, *Bmp6*, *Smad7*, *Tfr1*, and *Hfe* mRNA levels and LIP compared to the CT group. The association of tucum-do-cerrado with the iron-enriched diet (Tuc+Fe) decreased *Hamp*, *Hjv*, *Bmp6*, and *Hfe* mRNA levels and pSTAT3 protein content compared to the +Fe group, while increased *Hamp* and decreased *Hfe* mRNA levels compared to the Tuc group. Therefore, the inhibition of hepatic hepcidin by tucum-do-cerrado consumption may involve the downregulation of intestinal *Dmt1* and hepatic *Hjv* expression and deacetylation mediated by SIRT1 by a mechanism that is independent of tissue iron content. However, in excess iron conditions, the modulation of hepatic hepcidin expression by tucum-do-cerrado seems to be partially mediated by the inflammatory signaling pathway, as well as involves the chelating activity of tucum-do-cerrado.

## 1. Introduction

Iron constitutes an essential element for several metabolic processes in the human organism; both iron deficiency and excess iron may be harmful and lead to iron metabolism disorders [1,2]. Systemic iron homeostasis is finely regulated by the peptide hormone hepcidin, which is mainly synthesized by hepatocytes. Hepcidin inhibits duodenal iron absorption and iron release from macrophages and hepatocytes by binding to the iron exporter protein (ferroportin) and triggers its internalization and subsequent lysosomal degradation [3].

Hepatic hepcidin expression is modulated not only by hepatic and extracellular iron levels but also by other stimuli such as proinflammatory response, erythropoiesis, or hypoxia [4]. High iron levels and a proinflammatory profile improve hepcidin expression, while erythropoiesis and hypoxia inhibit it. In hepatocytes, transferrin receptor 1, transferrin receptor 2, and the human hemochromatosis protein (TFR1/TfR2/HFE), as well as the bone morphogenetic protein and hemojuvelin (BMP/HJV), constitute upstream regulatory proteins of two pathways that control hepcidin expression in response to circulating concentrations of transferrin bound iron (Tf-Fe_2_) and intracellular iron levels, respectively [3]. An increase in Tf-Fe_2_ levels displaces HFE from TfR1, which then interacts with TfR2, and the Tf-Fe_2_-HFE-TfR2 complex induces hepcidin transcription. BMP-6 protein levels are positively regulated by intracellular hepatocyte iron levels. BMP-6 and its coreceptor hemojuvelin interact with BMP receptors, which in their activated form induce SMAD 1/5/8 phosphorylation (p-SMAD1/5/8) and the consequent formation of the active transcriptional complex pSMAD1/5/8-SMAD4. The pSMAD1/5/8-SMAD4 complex translocates to the nucleus and upregulates hepcidin expression [1,3,5]. Interleukin-6 (IL-6) constitutes an upstream activator of hepcidin expression by the inflammatory signal transduction janus kinase (JAK)/STAT pathway. The binding of IL-6 to its cell surface receptor (IL-6R) actives JAK2, which in turn phosphorylates STAT3, and this subsequently dimerizes and translocates to the nucleus to induce hepcidin expression [1,3,5].

Besides these classical pathways, recently, some authors have described post-transcriptional modifications that may alter Hamp mRNA levels [6,7,8]. Xin et al. [8] showed that, in the presence of an inflammatory stimulus, sirtuin 1 (SIRT1) deacetylates the transcription factor STAT3, which in turn is deactivated, and Hamp mRNA transcription is inhibited.

Since the discovery of hepcidin and the identification of the proteins involved in the modulation of its expression, the identification of molecules that might act as agonists or antagonists of hepcidin have become a target for the treatment of disorders related to iron metabolism [9]. In a previous study by our group, we observed that the consumption of tucum-do-cerrado (*Bactris setosa* Mart.) inhibits hepcidin expression in rats even when associated with an iron-enriched diet [10]. Tucum-do-cerrado is a native fruit of Brazil with a high content of phenolic compounds [11] and antioxidant anti-inflammatory potential in vivo and in vitro [10,12,13]. Considering that the inhibition of hepatic hepcidin expression by tucum-do-cerrado might involve iron chelation by phenolic compounds and/or its antioxidant and anti-inflammatory capacity, we hypothesized that tucum-do-cerrado inhibits hepcidin expression through modulation of the TfR/HFE and/or BMP6/HJV/SMAD and JAK/STAT pathways.

## 2. Materials and Methods

### 2.1. Tucum-do-Cerrado Fruit Collection and Diet Preparation

Mature fresh tucum-do-cerrado fruits (*Bactris setosa* Mart.) were collected in the region of Terezópolis de Goiás, Goiás, Brazil (16_28015.4″ S and 49_03044.1″ W). After collection, fruits were transported immediately to the laboratory, washed in tap water, the seeds removed, and the pulp and peel kept at −80 °C for further use. The authenticity of the fruits was confirmed by UB Herbarium of the University of Brasília, Brazil. This study was approved by the Brazilian Institute of Environment and Renewable Natural Resources (IBAMA—license Number 9/2012). The diets were prepared monthly from the mixture of ingredients (Rhoster, Araçoiaba da Serra, SP, Brazil) following the proportion of ingredients proposed by Reeves et al. [14] and described by Fustinoni-Reis et al. [10], and stored at −20 °C. In the diets that contained the tucum-do-cerrado, the pulp and the peel were added as a homogeneous mixture to the other components of the diet.

### 2.2. Animals

Male Wistar rats, aged 25 days (75.0 ± 6.6 g), were purchased from Granja RG, São Paulo, Brazil. Rats were single-housed in a controlled temperature room (23 ± 2 °C) with a 12/12 h light/dark cycle. Before the experiment, rats were maintained for 4 weeks with AIN-93G diet [11] until they reached adulthood at two months old (248.9 ± 18.6 g). After this period, the 24 animals were randomly divided into four groups (six rats per group) and treated with one of the following diets: AIN-93G diet (Control/CT), which contained 35 mg of iron/kg of diet; tucum-do-cerrado diet (Tuc), i.e., the AIN-93G diet with the addition of 150 g of the edible parts (pulp and peel) of the tucum-do-cerrado fruit/kg of diet; the iron supplemented diet (+Fe), i.e., the AIN-93G diet containing 350 mg of iron/kg of diet; and the tucum-do-cerrado + iron supplemented diet (Tuc+Fe), i.e., the AIN-93G diet enriched with 350 mg of iron and 150 g of the edible parts of the fruit/kg of diet. The animals were weighed once a week, and food intake was recorded daily. At the end of 12 weeks of treatment, the rats were euthanized using previous anesthesia with 3% isoflurane followed by blood collection by cardiac puncture. The liver, spleen, gut, and kidney were excised, washed in cold 0.9% NaCl, and frozen in liquid nitrogen (N_2_), and the tissues were stored at −80 °C. The institutional Animal Care and Use Committee of the University of Brasília approved this experimental protocol (UnBDoc 20855/2014).

### 2.3. Determination of the Relative Amount of Labile Iron by Electron Paramagnetic Resonance (EPR)

The tissues were analyzed for labile iron using electron paramagnetic resonance (EPR) measuring the signal of the high spin Ferrioxamine (Fe^3+^ DFO) at g ~ 4.3 at T ~ 150 K [15,16,17].

Approximately 130 mg of tissue were homogenized using a mortar and pestle with 1.7 mL of PBS per gram of tissue weight. Then, 300 μL of DFO 10 mM per gram of tissue weight were added and the final volume of 500 μL was obtained with PBS supplementation. The homogenate was incubated for 1 h on ice and then frozen with liquid nitrogen in a syringe. The frozen homogenate was transferred to a liquid nitrogen finger dewar, which was inserted in the EPR spectrometer cavity. The EPR spectra were recorded using a Bruker EMX Plus spectrometer equipped with the X-band (ν ~ 9.45 GHz) high sensitivity cavity. The spectrometer parameters were: center field 1568 G, sweep width 1000 G, typical microwave frequency 9.4132 GHz, microwave power 50 mW, receiver gain 30 dB, modulation frequency 100 KHz, modulation amplitude 10 G, time constant 10 μs, conversion time 50.22 ms, and number of scans 5. The relative amount of labile iron for each sample was measured by the peak to peak intensity of the signal at g ~ 4.3 normalized by the mass weight of tissue. Each sample was made in triplicate and the results presented as mean values. We did not measure a Fe^3+^–DFO calibration curve and therefore we do not have the absolute number of Ferrioxamine centers. However, the spectrometer parameters were kept constant for all samples of each tissue and hence the intensities of the EPR signal of different samples of the same tissue are comparable and they are a measurement of the relative amount of labile iron.

### 2.4. Determination of the mRNA Levels

The extraction of total RNA from the liver and kidney was performed using TRIzol reagent (Invitrogen Inc., Carlsbad, CA, USA). Briefly, 100 μg of tissue were homogenized in 1 mL of TRIzol using a TissueRuptor (QIAGEN, Austin, TX, USA). After chloroform extraction, RNA from the aqueous phase was precipitated using isopropyl alcohol and dissolved in diethylpyrocarbonate-treated water. The RNA samples were precipitated using 3 mol/L (pH 5.2) sodium acetate (0.1× sample volume) and ethanol (2.5× sample volume), and this mixture was incubated at 4 °C for 30 min and then centrifuged at 10,000× *g* for 30 min at 4 °C. Ethanol (1 mL) was added to the pellet, and the sample was centrifuged at 10,000× *g* for 5 min at 4 °C, dried at room temperature, and diluted to 20 μL using deionized water. The RNA samples were quantified by measuring their absorbance at 260 nm, and their purity was assessed by calculating the absorbance ratios at 260:280 and 260:230 nm [18]. The integrity of the RNA was assessed by the electrophoretic profile on 1% agarose with 1000× Gel Green Nucleic Acid Gel Stain (Biotium Inc., Hayward, CA, USA) and a Tris/boric acid/ethylenediaminetetraacetic acid buffer solution, 1× gel (Sigma, St. Louis, MO, USA). Total RNA (1 μg) was used for complementary DNA (cDNA) synthesis reactions (20 μL final volume) using an ImProm-II Reverse Transcription System (Promega Corporation, Madison, WI, USA). OligodT primers were added to the total RNA, and denaturation reaction was carried out at 70 °C for 5 min. Improm-II Reverse Transcriptase was added, and the samples were incubated at 42 °C for 50 min, followed by inactivation at 70 °C for 15 min.

The mRNA concentrations of the hepcidin (*Hamp*), bone morphogenetic protein 6 (B*mp6*), hemochromatosis type 2 (juvenile) homolog (*Hfe2*), smad family member 7 (*Smad7*), transferrin receptor (*Tfr1*), hereditary hemochromatosis protein homolog (*Hfe*), divalent metal transporter 1 iron-responsive and non-iron responsive (*Dmt1* +IRE and *Dmt1* -IRE, respectively), and ferroportin (*Fpn*) were quantified using real-time polymerase chain reaction (PCR). This was performed using the Fast SYBR Green Master Mix 2× reagent (Applied Biosystems, Foster City, CA, USA) with 2.0 μL of cDNA (corresponding to 0.02 μg of total RNA) in a final volume of 10 and 5.0 μL of Fast SYBR Green Master Mix and 0.2 μmol/L (final concentration) of each primer (Table S1). Quantitative PCR was performed using a 7500 Fast Real-Time PCR System (Applied Biosystems) for 40 cycles at 95 °C for 20 s, 60 °C for 3 s, and 60 °C for 30 s. The amplification specificity of each amplified product was verified using a melting curve. The PCR amplification efficiency was evaluated by running standard curves for each amplicon in different template dilutions. A standard curve was plotted for each gene studied by correlating the ΔC_T_ (C_T_ target-C_T_ reference) versus the log of the cDNA dilutions in arbitrary units. A slope value of the regression line plot of ΔC_T_ values versus the log of input nucleic acid of <0.1 was used as a general criterion to accept the validation of the experiment, and a slope value of the regression line plot of C_T_ values versus the log of input nucleic acid of ~−3.32 was considered to be an efficient reaction. This method was performed as described in the tutorial “Guide to Performing Relative Quantitation of Gene Expression Using Real-Time Quantitative PCR” (Part No. 4,371,095 Rev B; Applied Biosystems). Relative gene expressions were calculated using the 2^−ΔΔCT^ method [19]. The results were normalized to the endogenous control, and the fold change of the gene expression was calculated using threshold cycle (C_T_) values. All samples were assayed in triplicates and were normalized to the housekeeping gene β-actin (Actb).

### 2.5. Immunoblot Analysis

An aliquot of the liver was homogenized in a buffer composed by 0.25 M sucrose, 15 mM Tris-HCl (pH 7.9), 15 mM NaCl, 60 mM KCl, 5 mM EDTA, 0.15 mM spermine, 0.5 mM spermidine, 0.1 mM phenylmethanesulfonyl fluoride (PMSF), 1.0 mM dithiothreitol, 1% protease inhibitor cocktail, and 1% phosphatase inhibitor cocktail (Sigma Aldrich, St. Louis, MO, USA), using a glass homogenizer. Samples were sonicated for one minute, centrifugated at 10,000× *g* for 10 min at 4 °C (Universal Refrigerated Centrifuge Z 326 K, HERMLE, Gosheim, Germany), and the supernatant was collected. The total protein concentration of the supernatants was determined using method described by Hartree [20]. Samples containing the same amounts of protein were separated in a 5−12% SDS-PAGE [18], and transferred to a 0.45 mm polyvinylidene difloride (PVDF) membrane (ImmobilonH-P transfer membrane-IPVH00010-Millipore-Billerica, MA, USA), using a semi-dry transfer system (Trans-Blot^®^ SD Semi-Dry Transfer Cell, BIO-RAD, Hercules, CA, USA). Membranes were blocked for 2 h in a shaker at room temperature in a buffer containing TBS/T 1× 0.5% nonfat dry milk. and then incubated with primary antibodies (diluted in 1× TBS/T + 0.5% nonfat dry milk) overnight at 4 °C. Different dilutions were used for each primary antibody (Table S2). Then, the membranes were washed three times with 1× TBS-T and incubated for 1 h with alkaline phosphatase-conjugated secondary antibody in a dilution of 1:1000 (# 7054-Anti-rabbit IgG, AP-linked Antibody, Cell Signaling, Danvers, MA, USA) and washed three times with 1× TBS-T. The intensity of immunoblotting bands was measured using BCIP^®^/NBT solution (B6404-Sigma Aldrich, St. Louis, MO, USA) and the ImageStudio Lite image analysis system (LI-COR Biosciences, Lincoln, NE, USA). β-actin was used as a loading control. The intensity of each immunoblotting band of the proteins of interest was divided by the intensity of β-actin band, and the results expressed in arbitrary units (A.U.).

### 2.6. Statistical Analysis

All groups were compared using the One-Way Analysis of Variance (ANOVA) test with Bonferroni correction. A *p* value of less than 0.05 was considered as statistically significant. Data are expressed as the mean ± standard deviation (SD). All analyses were done using the SPSS 17 software (SPSS Inc., Chicago, IL, USA).

## 3. Results

### 3.1. Effect of Tucum-do-Cerrado Consumption (Tuc) on the mRNA Levels of Hepatic Hepcidin (Hamp) and on the Gut Divalent Metal Transporter 1 Iron Responsive Element (Dmt1 +IRE), Divalent Metal Transporter 1 Non-Iron Responsive Element (Dmt1 -IRE), and Ferroportin (Fpn)

**Figure 1:**
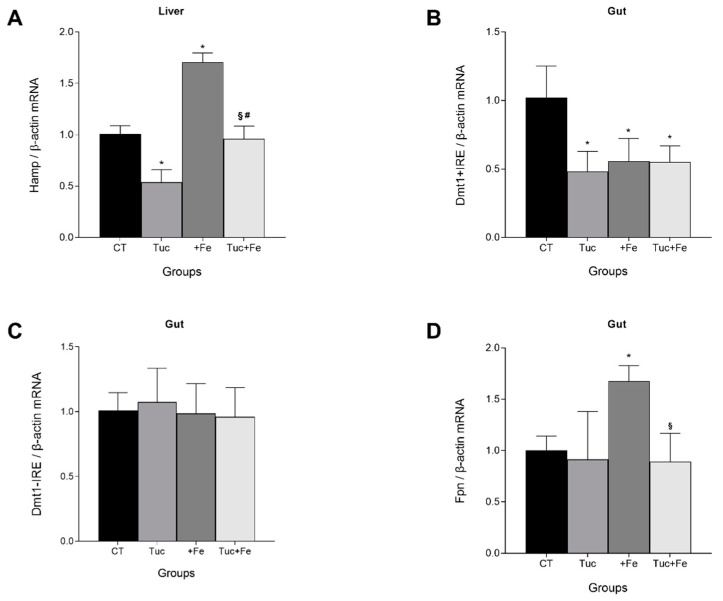
mRNA level quantification in rats fed control diet, tucum-do-cerrado (Tuc), iron-enriched diet (+Fe), or tucum-do-cerrado iron-enriched diet (Tuc+Fe), for 12 weeks, of: hepatic hepcidin antimicrobial peptide (Hamp) (**A**); and gut divalent metal transporter 1 iron responsive element (*Dmt1* +IRE); (**B**) divalent metal transporter 1 non-iron responsive element (*Dmt1* -IRE); and (**C**) ferroportin (*Fpn*) (**D**). Data are the average ± standard deviation (*n* = 6). Statistical differences compared to: * CT group; ^#^ Tuc group and ^§^ +Fe group and (*p* < 0.05).

To confirm that Tuc modulates Hamp expression even in a long-term treatment, we determined the hepatic Hamp mRNA levels after 12 weeks. Tuc promoted a decrease in hepatic Hamp mRNA levels compared with the control group in normal (Tuc) and iron supplemented (Tuc+Fe) rats (*p* < 0.001; Figure 1); however, the levels of Tuc+Fe group were higher than the values observed in the Tuc group (*p* < 0.001). The Tuc+Fe group showed lower hepatic Hamp mRNA levels than the +Fe group (*p* < 0.001) and similar to the control group (*p* = 1.000), while the +Fe group exhibited a 1.7-fold increase in the level of hepatic Hamp mRNA in relation to the control group (*p* < 0.001).

The intestinal absorption of iron is associated with Hamp expression. Therefore, the effect of Tuc on the intestinal mRNA levels of divalent metal transporter 1 with (*Dmt1* +IRE) and without iron-responsive element (*Dmt1* -IRE) were evaluated. Tuc promoted a 1.8-fold reduction in the Dmt1 + IRE mRNA levels, compared to CT (*p* = 0.003). Similarly, iron excess decreased *Dmt1* +IRE mRNA levels, even in the presence of Tuc (Tuc+Fe) in relation to the CT group (*p* = 0.002 and *p* = 0.001, respectively; Figure 1B). No difference was observed in gut *Dmt1* -IRE mRNA levels among any treatment (Figure 1C).

The mRNA levels of the iron exporter protein Fpn in the gut were also assessed (Figure 1D). Tuc did not alter the *Fpn* mRNA levels compared with CT; however, when associated with the iron-enriched diet (Tuc+Fe), the gut *Fpn* mRNA levels were reduced in relation to the +Fe group (*p* = 0.028). In the +Fe group, there was an increase of *Fpn* mRNA levels in relation to the CT group (*p* = 0.032).

### 3.2. Effect of Tuc on the BMP6/HJV/pSMAD 1/5/8 Pathway

**Figure 2:**
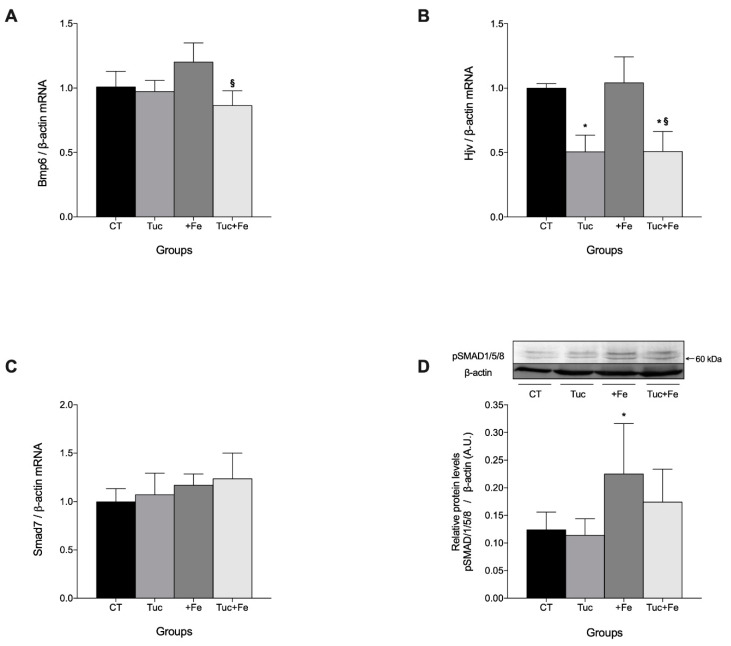
Hepatic mRNA level quantification of bone morphogenetic protein 6 (*Bmp6*) *(***A**), hemochromatosis type 2 (juvenile) homolog (*Hjv*) (**B**), and smad family member 7 (*Smad7*) (**C**) mRNA levels, as well as protein levels of pSMAD 1/5/8 (60 kDa) (**D**) in rats fed control diet, tucum-do-cerrado (Tuc), iron-enriched diet (+Fe), or tucum-do-cerrado iron-enriched diet (Tuc + Fe), for 12 weeks. Data are the average ± standard deviation (*n* = 6). Statistical differences compared to: * CT group, and ^§^ +Fe group (*p* < 0.05).

Next, to investigate whether the downregulation of Hamp mRNA by Tuc involves the BMP6/HJV/pSMAD 1/5/8 pathway, we determined the mRNA levels of these genes in the liver. Tuc did not alter hepatic *Bmp6* mRNA levels in normal (Tuc) and iron supplemented groups (Tuc+Fe); however, the rats exposed to both treatments (Tuc+Fe) showed lower *Bmp6* mRNA levels compared with the +Fe rats (*p* = 0.001; Figure 2A). Surprisingly, hepatic *Bmp6* mRNA levels in the +Fe group were similar to the control group.

Further, we observed that Tuc decreased hepatic *Hjv* mRNA levels in the groups treated with normal and iron-enriched diets (Tuc and Tuc+Fe) compared with the control group (*p* < 0.001 for both groups; Figure 2B). The Tuc+Fe group also had decreased hepatic *Hjv* mRNA levels relative to the +Fe group. No significant difference was observed in the hepatic *Hjv* mRNA levels of the +Fe group compared with the CT. Considering that Smad7 is another important inhibitor of hepcidin expression via Bmp/Smad signal transduction, the *Smad7* mRNA levels were evaluated in the liver, but no changes was observed among the groups (Figure 2C).

Considering that the hepatic hemojuvelin expression may potentiate the expression of hepcidin signaled by the BMP6 pathway, acting as a BMP6 co-factor, we further investigated the levels of phosphorylated SMAD1/5/8. Tuc did not affect the level of hepatic pSMAD 1/5/8, in either normal or iron-enriched diets (Tuc and Tuc+Fe). However, the +Fe group presented an increase in the levels of hepatic pSMAD1/5/8 relative to the control group (*p* = 0.045; Figure 2D).

### 3.3. The Effect of Tuc on the Tfr1/Hfe Pathway

**Figure 3:**
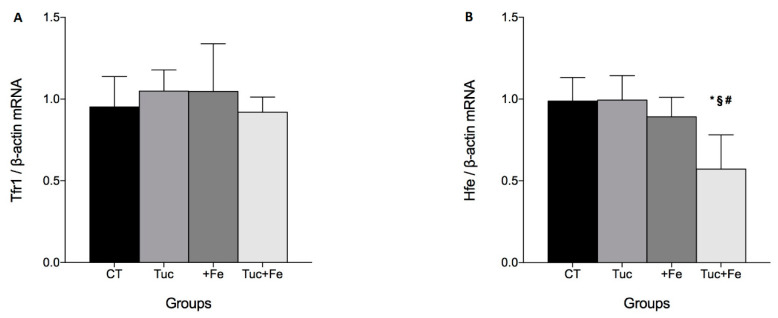
Hepatic mRNA level quantification of transferrin receptor (*Tfr1*)(**A**) and hereditary hemochromatosis protein homolog (*Hfe*) (**B**) mRNA levels in rats fed control diet, tucum-do-cerrado (Tuc), iron-enriched diet (+Fe), or tucum-do-cerrado iron-enriched diet (Tuc+Fe), for 12 weeks. Data are the average ± standard deviation (*n* = 6). Statistical differences compared to: * CT group; ^#^ Tuc group, and ^§^ +Fe group and (*p* < 0.05).

Given that Hamp expression is also regulated by Tfr1/Hfe pathway, we investigated the mRNA levels of *Tfr1* and *Hfe* in the liver. Tuc, iron supplementation (+Fe), and the combined treatment (Tuc+Fe) did not alter *Tfr1* mRNA levels in the liver (Figure 3A). Tuc did not alter hepatic *Hfe* mRNA levels in rats treated with normal iron diet; however, in the iron supplemented group (Tuc+Fe), it decreased *Hfe* mRNA levels compared to CT, Tuc, and +Fe groups (*p* = 0.002, 0.002, and 0.019, respectively). There was no difference in hepatic *Hfe* mRNA levels in the +Fe group compared with the control group.

### 3.4. The Effect of Tuc on the Hepatic pSTAT3 Protein Levels

Another stimulus that induces hepcidin expression is by the proinflammatory cytokines, which result in an increase of STAT3 protein phosphorylation. In another set of experiments with the same rats [10] (Figure S1), we observed that Tuc, in normal and iron excess (Tuc+Fe), did not promote any change in the levels of the proinflammatory markers IL-1β, IL-6, and TNF-α, compared with CT. However, the iron-enriched diet (+Fe) increased serum IL-6 and TNF-α protein levels, in relation to the CT (*p* = 0.028 and *p* = 0.024, respectively).

**Figure 4:**
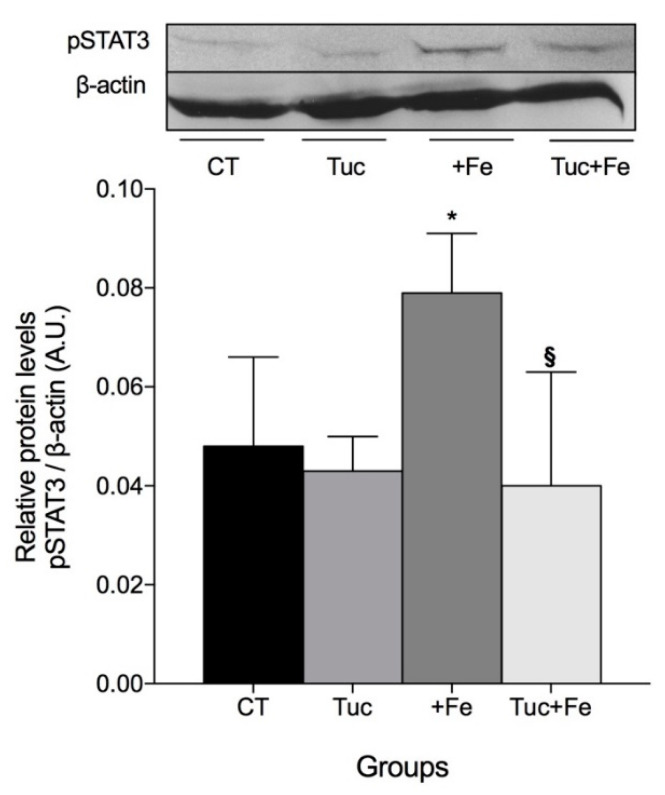
Hepatic pSTAT3 protein levels in liver of rats fed control diet, tucum-do-cerrado (Tuc), iron-enriched diet (+Fe), or tucum-do-cerrado iron-enriched diet (Tuc+Fe), for 12 weeks. Data are the average ± standard deviation (*n* = 6). Statistical differences compared to: * CT group; and ^§^ +Fe group and (*p* < 0.05).

Despite these results, Tuc significantly decreased hepatic pSTAT3 levels in rats treated with iron enriched diet (Tuc+Fe) in relation to +Fe group (*p* = 0.007). Iron supplementation (+Fe) promoted a significant increase in the hepatic pSTAT3 protein level compared to the control group (Figure 4).

### 3.5. The Effect of Tuc on the Labile Iron Pool of the Liver, Spleen, and Intestine

**Figure 5:**
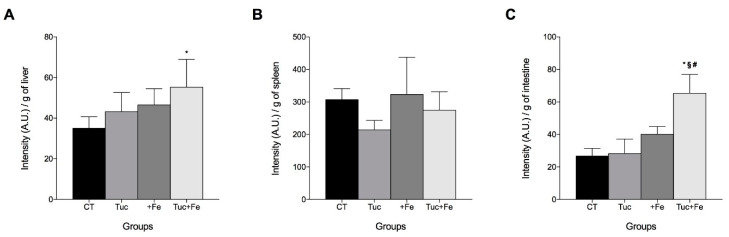
Labile iron pool levels of rats fed control diet, tucum-do-cerrado (Tuc), iron-enriched diet (+Fe), or tucum-do-cerrado iron-enriched diet (Tuc+Fe), for 12 weeks, in: liver (**A**); spleen (**B**); and intestine (**C**). Data are the average ± standard deviation (*n* = 6). Statistical differences compared to: * CT group; ^#^ Tuc group, and ^§^ +Fe group and (*p* < 0.05).

Our previous study showed that Tuc decreased Hamp mRNA levels in the liver of rats supplemented with iron, without any change in total iron concentration in the liver [10]. Therefore, we evaluated the tissues labile iron pool, to test the hypothesis that phytochemicals from tucum-do-cerrado decrease cellular iron availability, and thus decrease Hamp expression. Tuc did not alter hepatic labile iron pool in normal iron group (Tuc); however, in the iron supplemented group (Tuc+Fe), tucum-do-cerrado promoted an increase of labile iron pool compared to control group (*p* = 0.026; Figure 5A). No significant difference was observed in the +Fe group in relation to the control group. No significant difference was obtained in the labile iron pool in the spleen among any of the groups (Figure 5B). In addition, the Tuc+Fe group exhibited a higher labile iron pool in the gut than the CT, Tuc, and +Fe groups (*p* < 0.0001 for all comparisons).

## 4. Discussion

This study suggests that the downregulation of hepatic hepcidin mRNA levels promoted by Tuc seems to be mediated by *Hjv*, as both treated groups, Tuc and Tuc+Fe, showed lower *Hjv* and Hamp mRNA levels, compared with the control group. Unexpectedly, *Bmp6* mRNA levels and the phosphorylated SMAD1/5/8 (pSMAD 1/5/8) content did not differ in the Tuc and Tuc+Fe groups in relation to CT group. A previous study showed that exogenous *Hjv* introduced into *Hjv*-/- mice induced hepcidin expression without any significant change in mRNA levels of *Bmp6*, suggesting that hepatic HJV acts as the BMP co-receptor rather than promoting de novo BMP6 expression [21]. Contrary to our results, myricetin, a dietary flavonoid, decreased the hepatic hepcidin and *Bmp6* mRNA levels in a dose dependent manner [22]. However, the authors observed an increase in serum iron and a decrease in splenic iron in these rats, while in our study no difference was obtained in iron status. Contrary to what was observed in this study, a previous study from our group [10] showed a significant increase in *Bmp6* mRNA levels in the Tuc, +Fe, and Tuc+Fe groups compared with the control group. These conflicting results may be associated with the different treatment periods between the two studies (4 weeks versus 12 weeks). Despite the higher liver iron concentration, the treatment for 12 weeks caused an attenuation of liver iron increments among the different test groups when compared to the four-week treatment (e.g., 1.5-fold increment versus 1.8-fold increment, respectively).

Although high concentrations of iron in the liver seem to inhibit *Tfr1* expression, in the present study the iron-enriched diet (+Fe) did not alter hepatic *Tfr1* mRNA levels. Considering that transferrin saturation in the +Fe group remained higher than in the control group (40.80 ± 9.27 and 26.77 ± 3.76, respectively), we suggest that, with long-term iron supplementation, serum transferrin saturation remains slightly higher than control levels, while *Tfr1* mRNA must remain at normal levels, to allow the uptake and storage of excess iron by hepatic cells, and consequently recover the serum iron homeostasis.

The downregulation of hepatic hepcidin mRNA levels by Tuc seems not to be mediated by the iron TfR/HFE and the JAK/pSTAT3 inflammatory pathways, as the tucum-do-cerrado group (Tuc) presented similar *Tfr1*/*Hfe* mRNA and pSTAT3 levels in the liver compared to the control group. However, when Tuc was associated with excess iron conditions (Tuc+Fe), the downregulation of hepatic Hamp mRNA levels was partially mediated by the inflammatory pathway, as the Tuc+Fe group showed reduced levels of hepatic Hamp mRNA and pSTAT3 protein compared to the +Fe group. Considering that Tuc downregulated the hepatic hepcidin mRNA levels compared to the CT treatment, although both groups had similar levels of total iron in the tissues (liver, spleen, and intestine) and serum [13], we first hypothesized that the polyphenols present in the tucum-do-cerrado chelate intracellular labile iron, decreasing hepatocyte iron availability, which resulted in the inhibition of liver Hamp expression. Curcumin, a polyphenol derived from *Curcuma longa* (family Zingiberaceae), traditionally used in Indian medicine, is able to inhibit yeast growth by reducing intracellular iron availability [23]. Chin et al. also showed that curcumin downregulated hepcidin mRNA levels in mice, decreasing iron status by acting as a potent iron-chelator [24]. Contrary to our hypothesis and to the results observed with curcumin, in our study, Tuc combined with normal iron diet did not alter the labile iron content of tissues, suggesting that hepcidin inhibition by Tuc is mediated by a mechanism that is independent of tissue iron content. The similar levels of hepatic *Tfr1*, *Hfe*, and *Bmp6* mRNA, as well as of pSMAD1/5/8 protein, obtained in the Tuc and CT groups reinforces this hypothesis, as the modulation of hepcidin expression by iron levels is mediated by pSMAD1/5/8.

Although it is suggested that polyphenol-rich foods decrease iron availability due to their chelator properties [25,26], it is not yet established if other mechanisms may be involved. In our study, Tuc seemed to modify iron metabolism through the modulation of intestinal iron transport, as intestinal *Dmt1* mRNA levels were lower in the Tuc group in relation to the CT group and in the Tuc+Fe group in relation to the +Fe group. Despite the low expression of the transporter (*Dmt1*) responsible by iron uptake in the gut, no difference was obtained in total intestinal iron content, when comparing the Tuc with the CT group and the Tuc+Fe with the Tuc group. Similar to our results, Lesjak et al. [27] demonstrated that oral quercetin administration decreased duodenal *Dmt1* mRNA levels and did not alter duodenal iron content; however, they observed a decrease in mRNA levels of the iron export protein ferroportin. As expected, iron supplementation promoted the degradation of the *Dmt1* mRNA and activation of the *Fpn* mRNA translation in the gut, through the posttranscriptional IRE/IRP mechanism. This response was modified by the addition of Tuc to the iron-enriched diet (Tuc+Fe), as *Fpn* mRNA levels decreased compared to the +Fe group and remained similar to the CT group. The lower *Fpn* mRNA levels associated with the higher labile iron pool in the gut of the Tuc+Fe group suggests that the polyphenols present in tucum-do-cerrado exert a significant chelator affect in excess iron conditions. Therefore, iron metabolism appears to be modified by the chelating activity of Tuc as well as by its capacity to modify the expression of genes involved in iron absorption. In accordance with the literature [3], in this study, the induction of hepatic hepcidin gene transcription by the consumption of the iron-enriched diet (+Fe group) was mediated by both iron and inflammatory pathways, since hepatic pSMAD 1/5/8 and pSTAT3 protein levels were higher in the rats treated with the iron-enriched diet (+Fe) compared to the control rats. In fact, the rats from the +Fe group showed increased levels of serum IL-6 (data from other experiments conducted in these rats [13]; Figure S1), an upstream activator of hepcidin expression in the JAK2/pSTAT3 pathway. Even though consumption of the iron-enriched diet promoted an increase in hepatic pSMAD 1/5/8 levels in relation to the control group, no difference was observed in the mRNA levels of *Hjv*, *Bmp6*, *Tfr1*, and *Hfe* in the liver, which are proteins involved in the modulation of hepcidin by iron levels. Therefore, further protein level analyses are necessary.

Due to the anti-inflammatory properties of phytochemicals [28] and the modulation of hepcidin expression by the IL6/JAK2/STAT3 pathway, we next investigated whether tucum-do-cerrado inhibits hepcidin by promoting an anti-inflammatory response. Despite the high phytochemical content, Tuc did not alter the levels of the proinflammatory cytokine IL-6 (Figure S1) when associated with a control diet. In accordance with these data, no significant change was observed in pSTAT3 protein levels in the liver of the tucum-do-cerrado group compared to the CT group. Together, these results suggest that, in the absence of a proinflammatory stimulus, such as excess iron, the inhibition of hepatic hepcidin expression by tucum-do-cerrado is not mediated by the IL6/JAK2/STAT3 inflammatory pathway.

The capacity of tucum-do-cerrado to downregulate hepatic Hamp mRNA levels was partially maintained even with the consumption of the iron-enriched diet, as the Tuc+Fe group showed lower Hamp mRNA levels than the +Fe group and higher levels than the Tuc group. In excess iron conditions, tucum-do-cerrado seems to partially inhibit hepcidin expression by modulating the inflammatory signaling. The hepatic pSTAT3 concentration in the Tuc+Fe group was lower than in the +Fe group, and, even though serum IL-6 levels were not different between these groups, the Tuc+Fe group showed similar levels compared to the control group.

The increment in serum IL-6 in the +Fe group may also be associated with a potential modification in the gut microbiota, since iron supplementation increases the level of unabsorbed iron, which could affect the gut microbiome, favoring the growth of pathogenic strains and a proinflammatory response [29]. Jaeggi et al. [30] observed a shift in the gut microbiome of infants treated with iron supplements, showing a more pathogenic profile and an increase of intestinal inflammation. In accordance with this hypothesis and the inflammatory response observed in the Tuc+Fe group (described above), the polyphenols of tucum-do-cerrado may chelate excess iron when associated with iron supplementation (Tuc+Fe), which decreases iron availability to gut bacteria and consequently improves the microbiome profile and inflammatory response. Contrary to what was observed in the inflammatory pathway, the addition of tucum-do-cerrado to the iron-enriched diet (Tuc+Fe) did not alter hepatic pSMAD levels in relation to the +Fe group, suggesting that tucum-do-cerrado modulates hepcidin expression independent of the hepatic iron levels.

Recent literature suggests that hepcidin is also modulated by epigenetic events [7]. The administration of the pan-histone deacetylase inhibitor (pan-HDAC-panobinostat) abrogates hepcidin inhibition by iron deficiency or erythropoietin administration in mice. Similar results were also obtained in iron overload disease models, such as for β-thalassemia and hereditary hemochromatosis, suggesting that histone acetylation at the Hamp1 locus directly affects hepcidin expression [7]. Xin et al. [8] demonstrated that sodium hydrosulfide (H_2_S) treatment significantly suppressed LPS-hepcidin induction by promoting STAT3 deacetylation mediated by SIRT1. In the same study, the authors observed similar results when mice were treated with the polyphenol resveratrol, an inductor of SIRT1. Therefore, these data suggest that SIRT1 modulates hepcidin expression by deacetylation of STAT3.

In our study, the consumption of tucum-do-cerrado associated with a control or an iron-enriched diet significantly increased the mRNA and protein levels of SIRT1 in the liver of these rats compared to the control group (data from other experiments conducted in these rats [13]). Considering that the main catalytic activity of SIRT1 is the deacetylation of some histones [31], it is possible that one or more of tucum-do-cerrado’s phytochemicals improve SIRT1 activity, promoting the deacetylation of histones that interact with the hepcidin promoter region and consequently inhibit hepcidin expression.

Working with murine models of iron overload, Das et al. observed that resveratrol protects the liver from iron injury independent of hepatic iron levels [32]. This is similar to what was observed in our previous study, in which Tuc improved SIRT1 histone deacetylase protein levels [13]. Das et al. demonstrated that resveratrol increased SIRT1 levels and reduced the acetylation of the Forkhead fox-O-1 (FoxO1) transcription factor (FOXO1), promoting an increase in antioxidant gene expression [32]. Together, these findings support the hypothesis that tucum-do-cerrado inhibits hepcidin expression independent of the iron mediated pathway and suggest that it may involve deacetylation mediated by SIRT1.

## 5. Conclusions

The inhibition of hepatic hepcidin by tucum-do-cerrado consumption may involve the downregulation of intestinal *Dmt1* and hepatic *Hjv* expression and deacetylation mediated by SIRT1 by a mechanism that is independent of tissue iron content. Our data also show that in excess iron conditions the modulation of hepatic hepcidin expression by tucum-do-cerrado seems to be partially mediated by the inflammatory signaling pathway, aas well as involves the chelating activity of tucum-do-cerrado. Further studies are encouraged to investigate the potential of tucum-do-cerrado in epigenetic events and its relationship with iron metabolism.

## Supplementary Materials

The following are available online at https://www.mdpi.com/2072-6643/12/5/1515/s1, Figure S1: Typical EPR spectra of the homogenate, Figure S2: Hepatic mRNA levels quantification of interleukin 1 beta (*Il1b*) and tumor necrosis factor alpha (Tnfa) mRNA levels and serum protein levels of IL-1β, IL-6, and TNF-α in rats fed a control diet, tucum-do-cerrado (Tuc), iron-enriched diet (+Fe), or tucum-do-cerrado iron-enriched (Tuc+Fe) diet, for 12 weeks, Table S1: Primer sequences used for *Bmp6*, *Hamp, Hfe*, *Hfe2*, *Smad7*, *Trf1*, and *Actb* real-time PCR assays, Table S2: Dilutions and companies of primary antibodies used for protein immunoblotting.
