# Supplementary material for: The Action of JAK/STAT3 and BMP/HJV/SMAD Signaling Pathways on Hepcidin Suppression by Tucum-do-Cerrado in a Normal and Iron-Enriched Diets
Source: Nutrients. 2020 May 22;12(5):1515. doi: 10.3390/nu12051515 (PMC7285201; doi:10.3390/nu12051515)
Supplement: Supplementary file 1 [file nutrients-12-01515-s001.pdf]

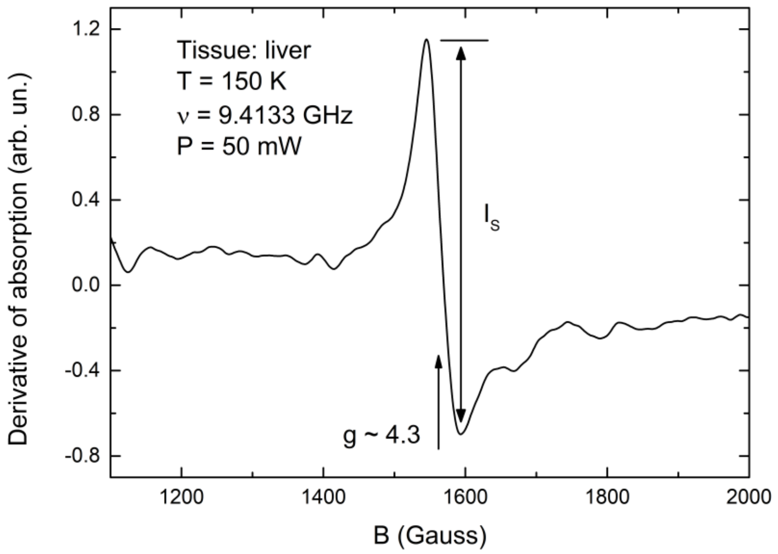

Figure S1. Typical EPR spectra of the homogenate. The arrows show the magnetic field for g = 4.3 and the peak to peak intensity (I_S) used to measure the relative amount of labile iron.

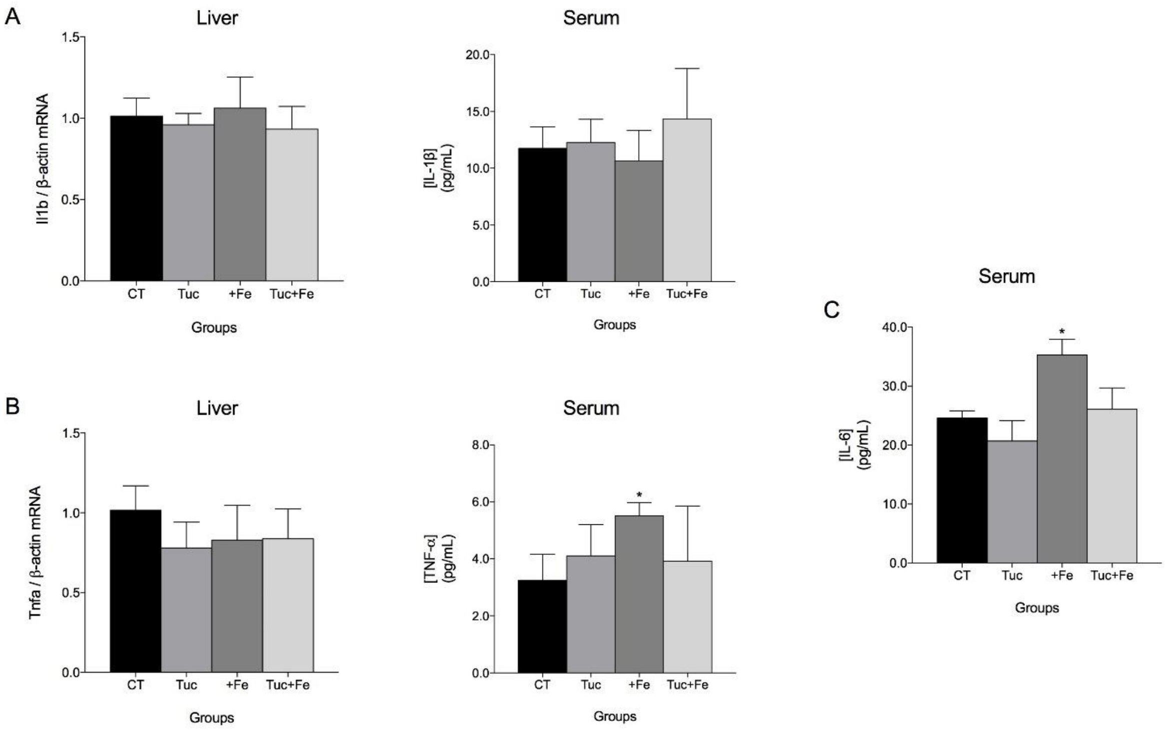

Figure S2. Hepatic mRNA levels quantification of interleukin 1 beta (Il1b) and tumor necrosis factor alpha (Tnfa) mRNA levels and serum protein levels of IL-1β, IL-6 and TNF-α in rats fed a control diet, tucum-do-cerrado (Tuc), iron-enriched diet (+Fe) or tucum-do-cerrado iron-enriched (Tuc+Fe) diet, for 12 weeks. Data are the average ± standard deviation (*n* = 6). Statistical differences compared to: * CT group, # Tuc group and § +Fe group ($p < 0.05$).

Table S1. Primer sequences used for Bmp6, Hamp, Hfe, Hfe2, Smad7, Trf1 and Actb real-time PCR assays.

Gene	Primers sequences (5'- 3')	GenBank accession number
**Bmp6**	GACAGCAGAGTCGCAATCG (forward)<br>AGCTCACGTAAAGCTCATGC (reverse)	NM_013107.1
**Hamp**	TGATGCTGAAGCGAAGGA (forward)<br>TGTGTTGAGAGGTCAGGAC (reverse)	NM_053469
**Hfe**	TGGGCAAGATCACCTTGAATT (forward)<br>GGATCCTGTGCTCTTCCCACT (reverse)	NM_053301.4
**Hfe2**	GTAGCATCGGGAGCCAAC (forward)<br>TCAAAGG CTGCAGGAAGATT (reverse)	NM_001012080.1
**Smad 7**	AGAGGCTGTGTTGCTGTG (forward)<br>CATCGGGTATCTGGAGTAAGG (reverse)	NM_030858.1
**Trf1**	GAGTTCACTGACATCATCAAGCA (forward)<br>TCCAGCCTCACGAGGAGTAT (reverse)	NM_022712.1
**Actb**	GTCGTACCACTGGCATTGTG (forward)<br>CTCTCAGCTGTGGTGGTGAA (reverse)	NM_031144

Table S2. Dilutions and companies of primary antibodies used for protein immunoblotting.

Protein	Dilution	Code/Company
pSMAD1/5/8	1:500	#9511 / Cell Signaling Technology
pSTAT3	1:500	#9131 / Cell Signaling Technology
β-actin	1:1000	#4967S / Cell Signaling Technology
